# Massachusetts Dental Society

**Published:** 1903-03

**Authors:** 


### MASSACHUSETTS DENTAL SOCIETY.

(Continued from page 121.)

### *Evening Session.*

*Dr. James Truman,* Philadelphia.—Mr. President, Ladies, and Members of the Massachusetts Dental Society,—The question that is before us to-night is one of vital importance to the dental profession throughout the United States and, may I add, throughout the world. It is a matter of regret to me that I am obliged to make an opposing stand to the generally expressed opinion on this subject, that law in connection with the practice of dentistry is of vital importance. My honest convictions lead me into the ranks of the opponents of this idea, as they have often led me in the past, to oppose majorities in other directions.

From the earliest dawn of civilization there has been a conflict under the name of law or its equivalent, custom on the one hand, and the spirit of liberty on the other, or, as Goethe expressed in Faust, evil and good always in conflict but finally good predominating, and this, I trust, may be the final verdict of the future histories of the world; although I confess to a pessimistic feeling in regard to the over-conquering power of good over evil. Good, if I read the pages of history aright, has always been victimized between two forces of evil,—law on the one hand, and bigotry, intolerance, and oftentimes brutality on the other. These two forces were found fully typified when the great Master of human thought was crucified between two thieves. In all ages these two are found side by side, with the good between, and antagonizing its efforts to uplift humanity. The inquisition of Spain represented law. The religious persecutions in England and Massachusetts were of the same character,—force, law, and bigotry.

Dentistry to-day stands between these two forces, and, as I view it, it is being sacrificed between these two that I must regard as evil.

Let us revert for a brief period to the history of the origin of law as applied to dentistry. The first law in this country was passed in 1841 in Alabama. There was a long period between the law of this State and the next to attempt the operation of force. This was in New York in 1868, followed by a State law in Ohio in the same year, and then in 1876 the State of Pennsylvania joined in the procession by an act regulating dentistry.

What were the conditions existing prior to the enactment of laws in these several States? From my own personal observation, the dentists of that period enjoyed a comfortable time. They were not troubled with law, and pursued their vocations with the kindly feeling that what they could do others had an equal right to perform. The man of empirical tendencies was there, but modest in comparison with the "Dental Parlor" man of to-day. They were so few and so comparatively unobtrusive that they were not a cause of much concern with the better men in dentistry. Competition was not great at that period, and the careful operator received the full value for his services. It was during this time of quiet professional life that some unrestful spirits demanded law to regulate the practice of the profession.

Who demanded that these laws should be enacted? Was it the good people who desired them? Were they praying our legislators to place these acts upon the statute-books of the several States, that they might be protected from the pretenders in dentistry? Did the demand for law come from the limited number of graduates of the period? Had the faculties of the dental colleges then existing any voice in the formation of these laws? Was it a demand from the dental profession as a whole? The answer to these several queries must be in the negative. Neither of these bodies expressed any burning desire for the enactmnt of laws. So marked was this indifference that legislatures after a time refused to pass the bills presented. They were unwilling to abridge the right of the people to earn an honest living. Who then did work for the laws? A few in the profession existing in all bodies and in all nationalities who have more faith in force than they have in progress by moral suasion.

Have these enactments, that are now on the statute-books of nearly every State of the United States, benefited anybody? Have they made the path easier for the students? Have they lessened the number of empirical establishments? Have they protected the people of the country? And, finally, have they protected the dental profession?

The argument has been advanced that through these laws and boards of examiners dental colleges have been forced to raise the standard of entrance, to increase the time and multiply the subjects of the curriculum. This never was true, for natural development has forced the dental colleges to an advanced educational position, and they would have reached this standard with or without law. The laws have made it more difficult for the student; inasmuch as he must pass a second examination at an increased and unnecessary expense.

The "Dental Parlors" have multiplied all over the land. While formerly confined to a few of the large cities, they have now a place in all small towns, lowering the standard and degrading the profession. By their alluring advertisements they tempt the people, and they, in the end, suffer, and the laws fail to protect them. Young graduates are tempted by good salaries to accept positions with these, and they, in turn, become ethically demoralized. I hold, therefore, that these laws, intended, it may be, for good, have proved dismal failures.

It seems to me that this craze for law, has been steadily growing, until the people of these United States can no longer call themselves free. Those who have lived in Europe can draw a lesson from the evil results of custom and law gradually depriving the people of liberty. From personal observation I am convinced that, let the form of government be what it may, whether a monarchy or nominally a republic, as in France or Switzerland, there will still exist the same depressing statutes. What is true there will in time be true here. It is the gradual and insidious encroachment of law over the freedom of the people. I do not wish to speak disrespectfully of law. I hold that it is necessary within limitations, but I am not willing to become a slave to a seemingly necessary evil.

I do not suppose that anything I may say here to-night will change the current of general thought on this subject or lead to a removal of a single law upon the statute-books. You are bound to be subject to law governing your profession. Hence, with every State seeking to make its law in medicine and dentistry more and more oppressive, it seems a waste of energy to meet this tendency with words. As a learned body of men and women you can, equally with myself, measure the results that are sure to come from this deification of force.

In closing, I propose to depart somewhat from the subject, as my friend on my right took the liberty of doing. Permit me first to thank you for the kind invitation to be present on this occasion, even in the disagreeable capacity of an opponent. I am comparatively a stranger to the majority here, but I am not a stranger to much that has made Massachusetts honorable among the people of this country. I have sat under the eloquence of Wendell Phillips, and have drawn inspiration from your Longfellow, Holmes, Whittier, and Lowell; have been taught wisdom by Emerson, and have gathered a liberal theology from Theodore Parker, and I can say with your great orator and statesman, Daniel Webster, "I can pass no encomiums upon Massachusetts, she needs none. There she is, behold her and judge for yourselves." She stands to-day the embodiment of all that is free in these United States. The spirit which animates her had its finest illustration amid the rattle of musketry at Bunker Hill, and found its deepest response at Independence Hall, Philadelphia, amid the acclaims of the multitude and the ringing sounds of the bell that proclaimed liberty throughout the land. It was echoed at Valley Forge and re-echoed at Yorktown, and when the other day I read a speech by a Massachusetts representative on the floor of the Senate of the United States, my soul was filled with admiration, and when I read his denunciation of my country for its work in the Philippines for its desecration of all that this people has regarded as sacred; when he denounced the horrors and brutalities committed by our soldiers in that country, only equalled by the Inquisition of Spain, I mourned for my native land, but gloried in the spirit of freedom that had not died in Massachusetts. If liberty be dead in the Philippines, the Anglo-Saxon race is preparing to bury the remains in South Africa.

Pardon this lapse into a foreign subject, and yet not altogether foreign, for it all concerns law on the one hand and brutality on the other. The spirit which antagonizes this has found its best life in this State, and may I repeat, Massachusetts! there she stands, behold her, the glory of the American Republic!

*President Faxon.*—It is not much of a task to introduce the next speaker. Do not misconstrue my meaning. It is not because he is of so little account, but because he is of so much account, so much consequence, holds such an honored position in the dental profession, as to be well-known and highly esteemed by all of you. I therefore take much pleasure and pride in introducing to you as the next speaker the Dean of the Dental Department of the University of Pennsylvania, and editor of one of the most valuable contributions to our dental literature, the *Dental Cosmos,* Dr. Edward C. Kirk.

*Dr. Edward C. Kirk,* Philadelphia.—MR. PRESIDENT, LADIES, AND GENTLEMEN,—I am not exaggerating when I say I am utterly at a loss as to how to proceed. I find myself very much in the position of the man who, I am informed, a short time ago went to a banquet without having been assigned a subject upon which to talk, and when he was called upon he rose with some embarrassment and said, "What shall I talk about?" Whereupon some one at the farther end of the room replied, "Talk about a minute." I feel very much inclined to talk "about a minute" or less. But seriously speaking, what can I say, what could be expected of me after the eloquent address of Dr. Truman? I might say that I

think those of us in the "majority" might pardon him for the heresies in the first portion of his address in consideration of the magnificent tribute which he has paid to the genius of Massachusetts in his peroration. Dr. Truman knows that any difference I may have with him in this connection or in any other is a difference of opinion only. If I am antagonistic, it is to what he has said and not to him personally. I must say frankly that I cannot agree with his position. As I view the matter,—and I know Dr. Truman believes I am honest in my convictions,—the law governing and limiting the practice of dentistry is not working out such evil results or such useless results or such fruitless results as he seems to feel that it is. I am not able, as he is, to say from personal experience what were the conditions governing dental practice at the time to which he refers, at the time previous to the enactment of law in dentistry, but I would like to add because of the impressions I have gained from my study of the historical aspects of our profession, if the conditions were as he has portrayed them, what were the causes which led to the formation of a national society of dentists at that time, or what were the conditions which led to the other great factors in our professional progress,—the founding of the first dental college and the first dental journal which marked our birth as a profession? These three great factors were established at the same time, bringing order out of a condition of professional chaos. It seems to me that the development of these three important factors was a practical protest against the charlatanism of the time. The unprofessional conditions of the time were thus brought under control to the end that a professional gathering such as this to-night might come into existence. Was it possible before that event to speak of a dental profession in any such sense as we have it to-day? I think not.

Now, let us examine for a moment another feature of this question of the dental law that has been touched upon somewhat by both of the gentlemen who have preceded me. The dental law is enacted for the protection of the public. That we know is its intent and purpose, and nothing else. Incompetence in the practitioner and professional advertising are by no means interchangeable terms. Dr. Truman makes a point that by reason of the operation of the dental law quacks are compelled to employ qualified men. Speaking in terms which are familiar to the distinguished jurist who has formally presented this case to-night,

I would say, may it please the court, it seems to me that when Dr. Truman admits that fact we may rest our case, for we have in that the demonstration of our main contention, because it is admitted than the law has compelled even the quack to render better professional service to the public by employing only qualified operators.

We find in the matter of dental legislation no relief from the annoying fact that a man may advertise and still render competent service to the public, and the law has not yet dealt with that question, nor will it ever be able to so long as advertising in general is regarded as a legitimate business procedure.

Now, as to the beginning of the dental law. There were no dental laws, strictly speaking, before there was a dental profession, but it is on record in the writings of the man who has been called the father of dentistry, Pierre Fauchard, that in the year 1700 a law provided that those who intended to practise dentistry in France should appear before a board of examiners to have their qualifications tested, and that they should not be permitted to practise until they obtained a license to do so. There was no explicit dental law until the law of Alabama was passed, but Horace Hayden practised dentistry under a license which was issued to him in 1810 by virtue of an enactment passed by the Legislature of Maryland in 1798 creating a Board of Physicians charged with the duty of examining into the qualifications of all who intended to practise medicine or surgery in any of its branches; so that the idea of dental legislature is not a novel one.

There are perhaps two other features to which I may be permitted to refer in this connection. First, with reference to our attitude as a profession towards the question of dental legislation. Our ideas in regard to it are necessarily the result of the evolution of our professional needs. It seems to me that it may throw some light on Dr. Truman's question if we consider the fact that a great deal of our trouble in connection with legislation depends upon our misunderstanding of the term "liberty." We will have to redefine the term liberty. It is true that by dental legislation we have placed a certain limitation upon something of a man's natural right to perform certain acts which in more primitive conditions of society would be properly regarded as an unwarrantable interference with his liberty. But as society is now organized the average common sense of the people says we may do this when one's natural right to do as one pleases is detrimental to the welfare of the community. It is not the individual's right to do altogether as he pleases, but as the community dictates he shall do without interfering with the equal rights of others, that constitutes liberty. If all men were willing to do the right, we would need no laws other than the ten commandments, but under existing circumstances we are compelled to prescribe limitations to the activities of certain classes in order that we may protect that thing which we call liberty and its enjoyment by the community. In the second place, it is quite true that much of our dental legislation is faulty and absolutely unjust, and I take it that the reason for that fact is to be found in the ignorance of the dental profession as a whole with respect to, first, what they want to accomplish by dental legislation, and, secondly, how far dental legislation is capable of meeting that want. We need further training and experience in this matter before we shall be competent to state just what ends we need to accomplish through dental legislation, and a still further and specialized training before we can accurately crystallize the expression of that need in the formal terms of a dental law which shall be equitable and just to all parties affected by it. Then there is the other obstacle to effective legislation, the outgrowth of the political entanglements which modify and control the actions of legislative assemblies. A bill may be correct and satisfactory in all of its provisions and yet fail of enactment as law by reason of the modifications and amendments to which it is often subjected by parties having conflicting personal interests which they bring to bear upon it in its devious course through the State Legislature.

I do not agree with Dr. Truman's idea that proper dental legislation has not been of value to our dental educational system. I believe that it has been of immense value. If the State requires a certain standard of professional qualification, and, more than that, prescribes that before entering upon his professional training the student shall have had a requisite amount of preparatory education, it seems to me that such a law necessarily acts as an influence to bring to the colleges men better prepared to profit by the college training; but further than that a high State standard of professional qualification is a check, and we may just as well acknowledge it right here, upon the tendency which here and there arises to graduate men who are not competent for the practice of dentistry. I am strongly of the opinion that in the fostering care of the State, expressed through the enactment of wise and equitable dental legislation, is to be found the principal hope for a high standard of dental education.

Then, if it is right and proper that the State should review and keep a check upon the educational work of the college faculties for the benefit of the public, it is also equally right and for the benefit of the public that the State should require an open public record of the work of the examining board. It is the public in both instances that are to be served, and the public that should be the final arbiter in both cases. Provision should be made for the prompt removal of any examiner upon proved charges of ignorance or any form of incompetency in his work. I am of opinion that when our conception of the desirability of a dental law which is equitable to all parties concerned has reached a point where we can formulate an act of that character, one which will conserve the right and eliminate the wrong from our system of educating and licensing practitioners of dentistry, all grounds for disagreement as between the teaching and the licensing bodies in dentistry will have been eliminated.

*President Faxon.*—Ladies and gentlemen, the next speaker on the programme has been so prominently before you through his eminent career that no words that I could say would add to your good opinion of him. I know you are all much more interested in what he is to say than in what I have to say about him. He is Dean of the Philadelphia Dental College, and I take great pleasure in introducing to you Dr. Guilford, of Philadelphia.

*Dr. Simeon H. Guilford,* Philadelphia, Pa.—Some one once asked the question, "Is life worth living?" and another wittily replied, "That depends upon the liver." And so it is, I think, with regard to the dental law. If one is asked whether he approved of the dental law, the reply will depend upon what particular dental law is under consideration, because, as we know, there are dental laws and dental laws; some are good, some are not. But there is one thing we are very certain of, and that is that the motives which have prompted the enactment of dental laws have always been good motives. There never has been anything back of the enactment of the dental laws that I know of that flavored of self-interest in any way; they were really intended for the good of the profession and the good of the public as well. It is unfortunate that we cannot have dental laws enacted in the different States that are considered good and yet are similar in character. It is due to the difference in human nature. Dental laws are not enacted by professional men, ordinarily so called, but by legislators, and I think even our legal friends will agree in saying that the ways of legislators are sometimes past finding out. Oftentimes we can accomplish something by approaching them in a certain way. If you would like to see a real "blue ribbon" legislator, come to Pennsylvania and we will show him to you.

But, as I have said, the opposition to dental law does not rest solely upon the fact that these dental laws are restrictive of the right of the dentists to practise. It is largely because of their imperfections. Let me give you an illustration. Suppose that in 1900 a young man living in the State of New Jersey, and who has already attended two annual courses in one of the registered medical colleges in New York City, desires to study dentistry. He decides to attend one of the dental colleges in Philadelphia. He does so and passes through with credit. He then thinks he would like to pass the Examining Board of Pennsylvania and receive his certificate, which he does. He returns to his home in New Jersey and there concludes to take the examination of the Board of New Jersey; he does so. About that time he is attracted to the city of New York, feeling that he will have a better opportunity to practise there. He sends on his credentials, and after a little while receives a paper stating that they are entirely satisfactory and that he will shortly receive a permit to go before the board and be examined.

Then let us suppose that in some way or other he fixes his eyes upon the State of Massachusetts, and thinks he should like to practise there. For some reason he decides that this would be better. He goes to Massachusetts, consults some member of the board, and is referred to the genial president of the State board, who tells him that it will be impossible for him to practise unless he takes an examination and pays a fee. He has already graduated from a reputable school in Pennsylvania, has taken the examination in Pennsylvania and New Jersey, with the promise of New York, and he wonders whether under the circumstances he cannot be admitted to practice in the State of Massachusetts. The officer informs him that it is quite out of order, and tells him very plainly that, while he is very sorry for him, the law requires that he should be examined. The young man says he has no particular objection to being examined, but he has already paid three examination fees in three different States, and he feels that it is hardly fair to ask a fourth. He is told, "If that is the case, there is nothing for you to do except to step out and retire." In other words, he must comply with the law.

He goes back to the State of New York to take his examination there. He finds a letter awaiting him stating that from his papers it would appear that he is not qualified to come up for examination, because, although he has spent two years in a medical college and two years in a dental college, the law requires three years in a dental college. The young man hurries off to Albany. He states to the regents that he has passed two years in a New York medical college and two years in a reputable dental college, and asks whether these four years of study are not enough to have given him sufficient qualification to register in the State of New York, especially as the National Association of Dental Faculties has declared that two years in a medical college shall equal one year spent in a dental school? The authorities say, "No; because the law requires three years in a dental school." He asks what he is to do. He is told, "The only thing for you to do is to go back to your college and attend another year. "But," says the young man, "I have graduated." "Never mind that, go back anyway."

He returns to New York City very much discouraged and disappointed. He retires and tries to sleep, but his brain is in such a whirl that he cannot sleep, and as he lies awake he thinks the whole subject over and wonders why it is that while he is in pursuit of a livelihood, a young man cannot pass from one State to another, and if he is qualified in two or three, why he should not be qualified in just as many more. He recalls that in Europe, when a man is declared qualified to practise in one city or part of France or Germany or Russia, he has the legal right to practise in all parts of that country, and being qualified in England entitles him to practise in any of her colonies as well.

That being the case, he does not understand why in free America, when he chooses to pass from one State to another, he has to be held up. He falls asleep and passes off to dreamland, and, as is so often the case with many of us, his last waking thoughts influenced and determined the character of his dreams. Translated to another sphere, he finds himself walking along the golden streets, and notices that the countenances of all whom he meets are perfectly serene and happy, and while he realizes that he should be as free from care or sorrow as they evidently are, he is conscious that he is not, and wonders why. Just then he meets a shade, which approaches him and says, "My friend, your countenance is sad and you seem sorrowful. Do you not know that the sphere in which you now are is one where sorrow and unhappiness are unknown, and that those who dwell here should be supremely happy? I noticed that as you walked the streets you would often glance around as though some fear oppressed you." The young man replied, "Perhaps I have done so unconsciously, because in the land from which I came I had been very much worried by individuals who have kept pursuing me and interfering with my pursuit of a livelihood by demanding fees and examinations." The shade replied, softly, "Rest thy soul, brother, those men never come here."

But that did not end the young man's troubles. After he waked he felt that there was nothing for him to do but return to his former home in New Jersey, which he did. After a number of years his health failed. He sought a milder climate in Colorado, but he found he could not practise in that State because he was not registered. Then he tried California, where he met with the same condition of affairs.

All of these misfortunes have probably not happened to one individual, but all of them have happened. I have tried to draw a composite picture, and have put the matter in this form that I might impress you with the fact that there are very grave weaknesses in our present dental laws. Take the State of Massachusetts. According to the law here, it is not necessary for a man to be a graduate of a dental college. All that is required is that he have sufficient education to enable him to pass an examination before the State board. The law works in this way. The man may receive a dental education in any way he pleases, and if he passes, well and good. But it happens that the examination is of such a character that he probably could not get through without some college training; so after two years in college he comes to Massachusetts and takes the examination before the board. If he should succeed, as he often does, there is no necessity for his going back to college; if he does not, he simply completes his college course and then comes back for examination. This is a radical weakness in the law of Massachusetts, and one which will have to be corrected. Another weak feature that belongs to all of the States is that a man who has been a reputable practitioner for a number of years cannot go into a neighboring State and practise dentistry without submitting to an examination. The man may have been very successful in practice, and have served his patients well; he may have kept fully abreast of his profession in a general way, or may even have been an instructor of students in a dental college, but he might not be able to pass a State board examination after his many years' absence from lectures and text-books. The older practitioners could not do it. I could not do it. I might pass in my own branch, but in nothing else. Another feature to which I desire to call attention is that while dental laws have been enacted for the protection of the general public, that is the very thing they do not do. Dr. Kirk makes the point that the dental parlors have been forced to serve the public better. Not a bit of it. They do not employ these young men because they are the best, but because they are graduates. They would not employ them for a moment if it were not that the laws cannot interfere with them. They are simply doing something to protect themselves, and they are demoralizing a class of young men who were intended for better and nobler things. These very men are themselves seduced, for they are taught while in college to respect and obey the laws. These proprietors of advertising offices are the very ones who should not be shielded by the law; and yet there seems to be no good way of preventing it. If our attorneys would get together and formulate a dental law which would take hold of these men and stop their illegal practice, we would all be grateful to them. If that could be done, dental laws in general would be more generally approved, for they would then be fulfilling their highest mission.

*President Faxon.*—We have three more speakers to divide the limited time among. I now have the pleasure of introducing to you Dr. Hurlbut, of Springfield, Mass.

*Dr. J. Searle Hurlbut,* Springfield, Mass.—MR. PRESIDENT, LADIES, AND GENTLEMEN,—I have listened with pleasure and pain to the remarks of the eminent speakers who have preceded me. With pain, because of some views here presented, and also that one by one they have taken up my subjects, till not one is left. I feel as might a fisherman who, equipped with rod, reel, and basket, about to drop the baited hook into the promising holes along the stream, suddenly discovers that others have just "worked the stream" and landed the fish. When I came here to-night, I thought the millenium had almost dawned for dentistry in this country. I had thought that the dental law was a power for good in elevating our profession, ranking with the colleges, the journals, and the societies in stimulating to higher education. In spite of assertions to the contrary heard here to-night, I think so still. Can it be that I have made sacrifices for these many years in vain, endeavoring to carry out the provisions of the dental law of Massachusetts, to the end that the people be protected and our profession elevated?

Only the opportunity for service to the people and to dentistry could have induced me to accept a position on the State board. On the passage of the law in 1887 the people were not in sympathy with it; even our profession within and without our societies gave it little support and some opposition.

Thus it behooved that commission to go slowly in enforcing the law. A sworn statement of having been in practice was all that was required to secure a license. The law could not be retroactive; could not deprive a man of his means of support. Doubtless many false returns were made, as the board had no authority to go back of such sworn statements.

In the examinations lower averages were accepted, until sentiment in favor of the law was strong enough to warrant the gradual increase that later followed. To aid the many impecunious applicants, the board often worked from nine in the morning till twelve at night, with but short rests at meal-times. Can it be that all this labor was in vain?

We have listened to-night to words of wisdom from an eminent barrister. He has given us his views, and his advice as his experience warrants. We have listened to words of wisdom from eminent teachers and journalists of our profession. Some have encouraged and cheered us on our progress. One grand sentinel of the past, a man much revered, honest, earnest, and beloved, has called us down from our high pedestal, and unfavorably contrasts the present with the past. Let the dead past bury its dead, say I. He truly says that "every State and Territory has its dental law." Does it not convince him, as it does us, that this unit of action means progress? Out of his own mouth is he condemned.

This is the twentieth century! The trend of our profession is upward.

Many points were brought out by these wise men from the South it were well to discuss, but the lateness of the hour prevents.

In many States a diploma from a reputable college admits to practice. Not so in Massachusetts: the law expressly states that "applicants must be examined." The wisdom has been apparent many times. The same conditions are enforced in the legal profession. The last speaker said, "It is a hardship not to have an interstate license." In many cases it is, but in the main it is just and right. As "no chain is stronger than its weakest link," so the standard of practice would be lowered, the incapables would gravitate to the "easy" States for examination, and thus obtain permit to practise in States where they previously failed.

Not till a national commission shall do all the examining, or all the States have uniform examinations, would it be wise to make the change. The rightful solution of this question is foreshadowed in the acts of all progressive colleges, requiring a high school education and four years for graduation. Thus equipped, a candidate would be able to pass any and all State boards.

I will detain you but a moment longer, to point to a weakness in our own State law, in its lack of means to prosecute offenders. I would have our State society appoint a prosecuting committee with full power to engage legal talent or other agents. All surplus moneys turned by the board into the State treasury should be available for this purpose. If necessary, the State society should appropriate a further sum. Ladies and gentlemen, I thank you for your courtesy.

*President Faxon.*—Dental legislation is still undeveloped, and I know of no one who is better able to carry on this discussion than one who has been a member of the Board of Dental Examiners of the State of Connecticut, Dr. George L. Parmelee.

*Dr. George L. Parmelee,* Hartford, Conn.—Most examining boards have their practical and their theoretical examinations. Occasions like this, too, have their practical and theoretical side, and I have been hoping all the evening that you would only expect me to do my duty on the practical side,—that is, to give you a practical demonstration at the table. In performing this practical duty I feel that the most tyrannical examiner would give me ninety-five per cent., which will, I trust, help me through the theoretical ordeal I am now about to attack.

I have always been a firm believer in the theory that the way to a man's heart lay through his stomach, my favorite proverb being, "The carving knife is mightier than the sword." I have been and still continue to be a staunch advocate of the social feature at all professional meetings.

A great deal of effort has been rightly expended, first and last, in endeavoring to obtain such legislation as should elevate and purify the practice of dentistry, but I am inclined to think that the influences of such an organization as yours,—and occasions like this,—Mr. President, may exert more lasting benefit along those lines.

For me to attempt to address you after the illustrious guests that have preceded me seems presumptuous, or, as the Japanese say, "Like painting feet on snakes."

The field covered by the subject under discussion this evening is so vast that I feel very much as did one of your little Massachusetts school-boys. His teacher, after telling her class all about the landing of the pilgrims, requested her pupils to draw from imagination a picture of the Plymouth Rock. There was considerable confusion, however, when one little fellow got up and asked if she wanted a hen or a rooster.

The majority of the dental world labors under the idea that the dental laws were passed to protect the dentist from the dentist; that is to bar out the "dental parlor" man and admit to practice only those who live up to the code of ethics. Still, notwithstanding the fact that more or less selfishness on the part of the dental profession prompted the advocacy of such enactments, the laws as they exist to-day are for the safety and welfare of the people.

Where the successful prosecution of a calling requires a certain amount of technical knowledge and professional skill in a practitioner, and where the lack of it may result in damage to one who employs him, it is a legitimate exercise of police power to prohibit any one from engaging in such calling who has not been previously examined by lawfully constituted authority and received a certificate of qualification.

The writer of a recent article in one of our journals severely criticises our State boards because they have not diminished the number of dental parlors.

Every class of society has its scum, and our specialty is certainly no exception. I favor a high standard for license, but a high standard, while it demands competence in those entering upon practice, cannot control their future actions or methods of practice.

Examiners cannot, other things being equal, discriminate, even though they are convinced that the candidate will practise in utter violation of the code.

The law does not and will not dictate methods of practice, but simply expects the examiners to determine whether or not the candidates are safe persons to treat the teeth of the public. The question of conduct of practice—or malpractice—is another story.

What do the class of people who patronize the dental parlors care for the code of ethics? From their stand-point, the man who apparently gives them the most for their money is the best dentist. They do not want to be our patients; we do not want them.

If the examiners see to it that only competent men enter practice, these offices could, if they only would, do a good work among the poor. Such offices are better in some respects than the pauperizing dispensary evils of some of our large cities.

The advertising man is the dentist of the masses, and can, if he will, do well by them. Dental legislation can never force the masses to patronize only code-abiding men, neither can it prevent the best-educated practitioner from being a quack if he so desires.

"You can lead a horse to water, but you cannot make him drink." So too, you can educate a dental student and deluge his mind with all that is best in life, but when he has graduated, unless he has in his heart a true conception of the Golden Rule,—which is the germ of all ethical codes,—your labors have been in vain.

Regarding the mutual relations of the State dental societies and the examining boards it would hardly seem possible that there could be any question. The dental societies should represent the best that is in dentistry, in the community, and should have at least the privilege of submitting a list of names to the executive from which the examiners are chosen and appointed.

The problem as to how we can best combat the evils of political influence and, worse than all, the petty "peanut politics," rings, and boss rule existing in some societies, remains to be solved.

On general principles, then, I claim that the examiners and the societies should in all ways possible aid each other.

Now a word regarding the preliminary training of the dental student. It is not for me to say how dentistry should be taught. Let abler minds attend to that; but thirty years' experience in practice has firmly impressed upon me that the best preliminary training for any man is that which will best fit him for his life's work. Therefore, for the dentist I would suggest that his literary training be ample; that he have such thorough grounding in physics and biology as to insure a firm foundation on which to build his special science; but first of all, for a dental preliminary, let him acquire the manual training "whereby hand, mind, and eye are educated by means that conserve vitality and develop a union of thought and action."

I am fully convinced that some method must sooner or later be devised to bring about an interchange of licenses between States. Still I have heard some able arguments against such interchange, as tending to create a roving spirit, and as being of greater value to the itinerant practitioner than to any other.

The New England Association of Dental Examiners has been, and still is, doing good work along these lines, and it seems to them that the only method whereby the interchange can be accomplished is by the gradual equalization of the standards of examinations in adjoining States.

We may get the best dental laws passed, but unless there are better means of enforcing them than there seem to be at present, the benefit to be derived from them is limited.

Here, too, there seems to be a mistaken impression in the dental world that on the examiners alone devolves the duty of prosecutions.

The ethics of their profession and official position may justify and possibly expect from them greater interest in securing the faithful observance of these especial laws than would be expected from the ordinary citizen, but the extent to which they shall gratuitously sacrifice themselves, their own time, money, or convenience, is for their own judgment to decide.

The examiners should be ready at all times to extend every possible aid and information in such cases. Collecting evidence and acting as prosecuting agents are not within their province.

While it is evidently the duty of every well-disposed citizen to see that the law is obeyed and to assist in securing evidence, it is a well-known fact that what is every man's business is no man's business, and is never accomplished.

The dental societies of a State should take up this duty, appoint prosecuting committees, and establish a fund sufficiently large to thoroughly accomplish the task. I am correctly informed that in a near-by State—and, in fact, about the only State where the matter of prosecution is fully attended to—one of the examiners has expended several thousand dollars in the work from his own private funds. Unfortunately, all examiners are not in position to do this.

*President Faxon.*—A few moments before we came into the banquet the next speaker asked me, "Why did you put me at the end of the list?" My answer was, "I put you there because I knew you had the ability to say a great deal in a short time, and we want some one with that ability to wind up the evening, and, therefore, you were chosen." Allow me, therefore, to introduce to you Dr. Thomas Fillebrown, of Harvard University.

*Dr. Thomas Fillebrown,* Boston, Mass.—MR. PRESIDENT, LADIES, AND GENTLEMEN,—I suppose I am called upon to pronounce the benediction. At this late hour it ought to be short. This whole subject has been discussed so thoroughly that there is little left for me to say. I can certainly say "Amen" to the most that has been said to-night. While I disagree with our friend from Philadelphia, I acknowledge that there is a good deal of truth in the spirit of what he says. I fully agree with the remark of my friend Guilford, that the inefficiency of the laws comes from the neglect of their enforcement. This is because human nature has not become quite perfect enough yet to work out its own salvation in the true way. I think it might interest our Philadelphia friends and the strangers here to-night for me to recall the history of the dental legislation here in Massachusetts. Several times before this law was passed this Society sent a committee to the Legislature asking for a dental law, and it resulted in simply nothing at all. The committee could not obtain even a patient hearing. Finally, Dr. Lewis T. Foss, of this city, single handed, without assistance, financial or otherwise, went to the Legislature, and during the same session Governor Ames signed a dental registry bill. This same man was my assistant for some years, so, of course, I am a remote cause of the result.

There is one phase of the law that has not been discussed to which I will call your attention. It is the administration of the law that makes law beneficial to the community. In every community violations of law are winked at. Occasionally it may be for the best interests of the public that this is so in isolated cases. Perhaps there are some laws here in Massachusetts administered in some such way. It is the duty of the community, the profession, and society in general to see that the law is properly administered. A dental law must be obeyed in every case if it is to be respected. Every person who is interested in our State societies should see to it that men are appointed to the Board of Dental Registration of Massachusetts that are free from political obligations, and as far as possible of superior learning and professional skill, unprejudiced and of broad judgment, so that we who are devoting our time, energy, and money to the education of young men can feel that we have a standard which is pure, high, and, above all things, impartial. We as teachers should also have the ability to make them feel that our judgment is something they can rely upon. Now I give you my blessing.

Adjourned.

(To be continued.)

